# Clinical efficacy of the laparoscopic modified Soave procedure for Hirschsprung's disease: a comparative retrospective cohort study

**DOI:** 10.3389/fped.2025.1700323

**Published:** 2025-12-19

**Authors:** Chuncan Ma, Yong Li, Yalan Xia, Xintao Zhang, Qiongqian Xu, Xixi He, Dong Sun, Jichang Han, Xue Ren, Dongming Wang, Jian Wang, Aiwu Li

**Affiliations:** 1 Department of Pediatric Surgery, Qilu Hospital of Shandong University, Jinan, China; 2 Fifth Department of General Surgery, Xiangxi Autonomous Prefecture People’s Hospital (First Affiliated Hospital of Jishou University), Jishou, China; 3 Second Department of General Surgery, Hunan Children’s Hospital, Changsha, China; 4 First Department of Cardiovascular, Xiangxi Autonomous Prefecture People’s Hospital (First Affiliated Hospital of Jishou University), Jishou, China

**Keywords:** laparoscopic modified Soave procedure, Hirschsprung's disease, clinical efficacy, retrospective cohort comparative study, transanal Soave procedure

## Abstract

**Background:**

Hirschsprung's disease (HSCR) is the second most common congenital gastrointestinal malformation, posing a significant health concern in pediatrics. The laparoscopic modified Soave procedure, a minimally invasive technique, has gained popularity due to its potential advantages. This study aimed to evaluate its clinical efficacy in comparison with the traditional transanal Soave procedure.

**Methods:**

This comparative retrospective cohort study included children with HSCR treated at Qilu Hospital between January 2014 and January 2024. The patients were divided into the following two groups: the Laparoscopic group (those who underwent the laparoscopic modified Soave procedure) and the Transanal group (those who underwent the transanal Soave procedure). The assessed outcomes included postoperative recovery metrics, complication rates, and 1-year follow-up results.

**Results:**

In total, 96 patients were included in the study. Compared with the Transanal group, the Laparoscopic group demonstrated reduced surgical time, faster gastrointestinal recovery, and reduced hospital stay duration (*P* < 0.05). Intraoperative blood loss was greater in the Laparoscopic group (*P* < 0.05). Complication rates were lower in the Laparoscopic group (4.17%) than in the Transanal group (14.58%), although the difference was not statistically significant (*P* > 0.05). Notably, the incidence of postoperative abdominal distension was lower in the Laparoscopic group (*P* < 0.05), but no significant differences observed in multivariate analysis of postoperative outcomes (*P* < 0.05).

**Conclusion:**

The laparoscopic modified Soave procedure demonstrated superior clinical efficacy compared to the transanal approach, offering faster recovery and a trend toward fewer complications. These findings support its wider adoption as a minimally invasive treatment option for HSCR.

## 1 Introduction

### 1.1 Research background

Hirschsprung's disease (HSCR) (1) is a congenital gastrointestinal malformation with an incidence of approximately 1 in 5,000 live births, making it the second most common congenital anomaly of the digestive tract. Clinically, HSCR presents with symptoms such as chronic constipation, persistent abdominal distension, and episodes of acute intestinal obstruction. Pathologically, it is characterized by the absence of ganglion cells in the submucosal (2) and myenteric plexuses of the intestinal wall, resulting in tonic contraction of the aganglionic segment, impaired peristalsis, and accumulation of intestinal contents (3). This leads to progressive dilation of the proximal bowel and poses significant health risks for affected children.

Although stem cell therapy has emerged as a promising direction for HSCR treatment in recent years (4, 5), surgical resection remains the mainstay of clinical management. The standard procedure involves the excision of aganglionic segments and the pull-through of normally innervated bowel to restore functional continuity and bowel motility.

With ongoing advances in pediatric surgery, minimally invasive techniques are increasingly favored due to their advantages of reduced surgical trauma, faster recovery, and superior cosmetic outcomes. Laparoscopic approaches, in particular, have gained widespread acceptance in the treatment of various pediatric gastrointestinal disorders and have shown considerable promise in the management of HSCR (6).

### 1.2 The evolution of the Soave procedure

The surgical management of Hirschsprung disease has progressed significantly since Swenson performed the first pull-through procedure in 1948. In 1964, Soave introduced the abdominoperineal submucosal rectal muscle sheath pull-through, which preserves perirectal neurovascular structures and minimizes sphincter injury. Boley later refined this into a single-stage procedure in 1968, thereby reducing surgical risks.

A major advancement occurred with the transanal approach, pioneered by So et al. (7) in 1980, and this was subsequently established as a fully transanal pull-through by De la Torre et al. (8) in 1998. Georgeson's introduction of the laparoscopic-assisted technique in 1995 further advanced this procedure, making it a minimally invasive approach.

These developments have largely replaced open approaches, resulting in reduced postoperative pain and shorter recovery times. Technical refinements in submucosal dissection and muscle sheath management have decreased complications such as anastomotic stenosis and Hirschsprung-associated enterocolitis (HAEC). The ongoing evolution of the Soave procedure reflects a sustained emphasis on functional preservation and improved clinical outcomes.

### 1.3 Research purpose

The Soave procedure has evolved through iterative refinements emphasizing minimally invasive strategies, anatomical preservation, and complication reduction. Its enduring relevance in pediatric surgery underscores the collaborative progress in HSCR management. The laparoscopic modified Soave procedure offers distinct advantages, including enhanced intraoperative visualization, reduced blood loss, and minimized scarring (9, 10). These benefits are particularly critical in pediatric populations, where rapid recovery and reduced trauma are paramount (11, 12). However, it is argued that the incidence of postoperative complications after transanal surgery is lower.

This study aimed to evaluate the clinical efficacy of the laparoscopic modified Soave procedure in the treatment of HSCR and compare it with the transanal Soave procedure.

## 2 Materials and methods

### 2.1 Statement on compliance with standards and ethics

This comparative retrospective cohort study was registered on a public clinical trial platform. The structure of this article follows the STROBE (Strengthening the Reporting of Observational Studies in Epidemiology) guidelines to ensure transparency and completeness in reporting. Ethical approval was obtained from the Ethics Review Committee of Qilu Hospital, Shandong University (Approval Number: KYLL-202503-051). This was a retrospective study involving human participants, and ethical approval (No. KYLL-202503-051) applies to this work. As all the data were collected from electronic medical records, informed consent was waived. This study was conducted in accordance with relevant ethical standards and regulations, including the Declaration of Helsinki.

### 2.2 Study design

This comparative retrospective cohort study included children diagnosed with HSCR who were admitted to the Department of Pediatric Surgery at Qilu Hospital between January 2014 and January 2024. The participants were divided into the following two groups: the Laparoscopic group, comprising participants who underwent the laparoscopic modified Soave procedure, and the Transanal group, comprising those who underwent the transanal Soave procedure. Clinical outcomes, including postoperative recovery metrics, complication rates, and results from a 1-year follow-up, were compared between the two groups to assess the relative efficacy of each surgical method.

### 2.3 Outcome and definitions

**Outcome:** The follow-up period was 1 year. The outcome was defined as the occurrence of any complication of concern during the follow-up period, or the absence of complications by the end of follow-up.

Definitions of relevant indicators:

**Enterocolitis:** The classic manifestations of HAEC include abdominal distention, fever, diarrhea, and other signs or symptoms, which may include vomiting, rectal bleeding, lethargy, loose stools, and obstipation (13).
**Wound infection:** The wound site may show local redness, swelling, warmth, pain, and increased secretions that may be purulent. Systemic symptoms such as fever may also be present.
**Intestinal obstruction:** Patients may present with abdominal pain, abdominal distension, vomiting, and cessation of flatulence and defecation. Abdominal pain is typically paroxysmal colic. Abdominal distension may worsen as the obstruction persists. Vomiting frequency and content vary depending on the site of obstruction.
**Gastrointestinal recovery time:** Defined as the time to the first anal discharge (passage of gas or stool) after surgery.

### 2.4 Data collection

Data were extracted from hospital medical records, with the patients in both groups having short-segment HSCR for surgical treatment.

The inclusion criteria were as follows: (1) diagnosed with HSCR by barium enema or rectal mucosal biopsy; (2) presentation of typical clinical manifestations such as constipation and abdominal distension; (3) patients with short-segment HSCR; and (4) underwent laparoscopic modified Soave radical surgery or the transanal Soave procedure in the pediatric surgery department of Qilu Hospital.

The exclusion criteria were as follows: (1) patients over 18 years old or those who underwent their initial surgery at another hospital; (2) patients with organ dysfunction or other congenital anomalies; (3) patients with syndromic HSCR or a history of necrotizing HAEC; and (4) patients with concomitant digestive system disorders, severe endocrine diseases, or acute and chronic infections.

Before the operation, we thoroughly explained the advantages and disadvantages of these two surgical methods to the parents and let them choose one of them. The management plans for the two groups of patients after the operation were essentially the same and there were no significant deviations over time.

### 2.5 Surgical procedure

#### 2.5.1 The abdominal phase of the laparoscopic modified Soave procedure

All the patients in the Laparoscopic group underwent daily saline colonic irrigation starting 7 days before surgery to ensure bowel cleanliness and reduce the bacterial load. A strict fasting protocol and intravenous fluid management were initiated 24 h prior to the surgery to maintain fluid balance and allow for the administration of prophylactic antibiotics. The modified Soave laparoscopic procedure was performed under general anesthesia.

A curved incision was made 1 cm below the umbilicus, followed by the insertion of a 5-mm trocar to establish pneumoperitoneum (carbon dioxide pressure was maintained at 10–12 mmHg for patients aged 1–10 years). Two additional 3-mm trocars were symmetrically inserted at the midclavicular lines on both sides of the umbilicus. The abdominal cavity was systematically inspected using a 30° laparoscope, with careful identification of key anatomical landmarks to prevent iatrogenic injury.

The level of intestinal resection (aganglionic segment, transitional segment, or dilated segment) was determined based on preoperative barium enema findings, intraoperative frozen section analysis of the aganglionic segment, and the degree of bowel dilation. The aganglionic, transitional, and dilated segments of the intestine were mobilized and resected. Hemostasis was achieved by ligating the relevant blood vessels.

**Figure 1:**
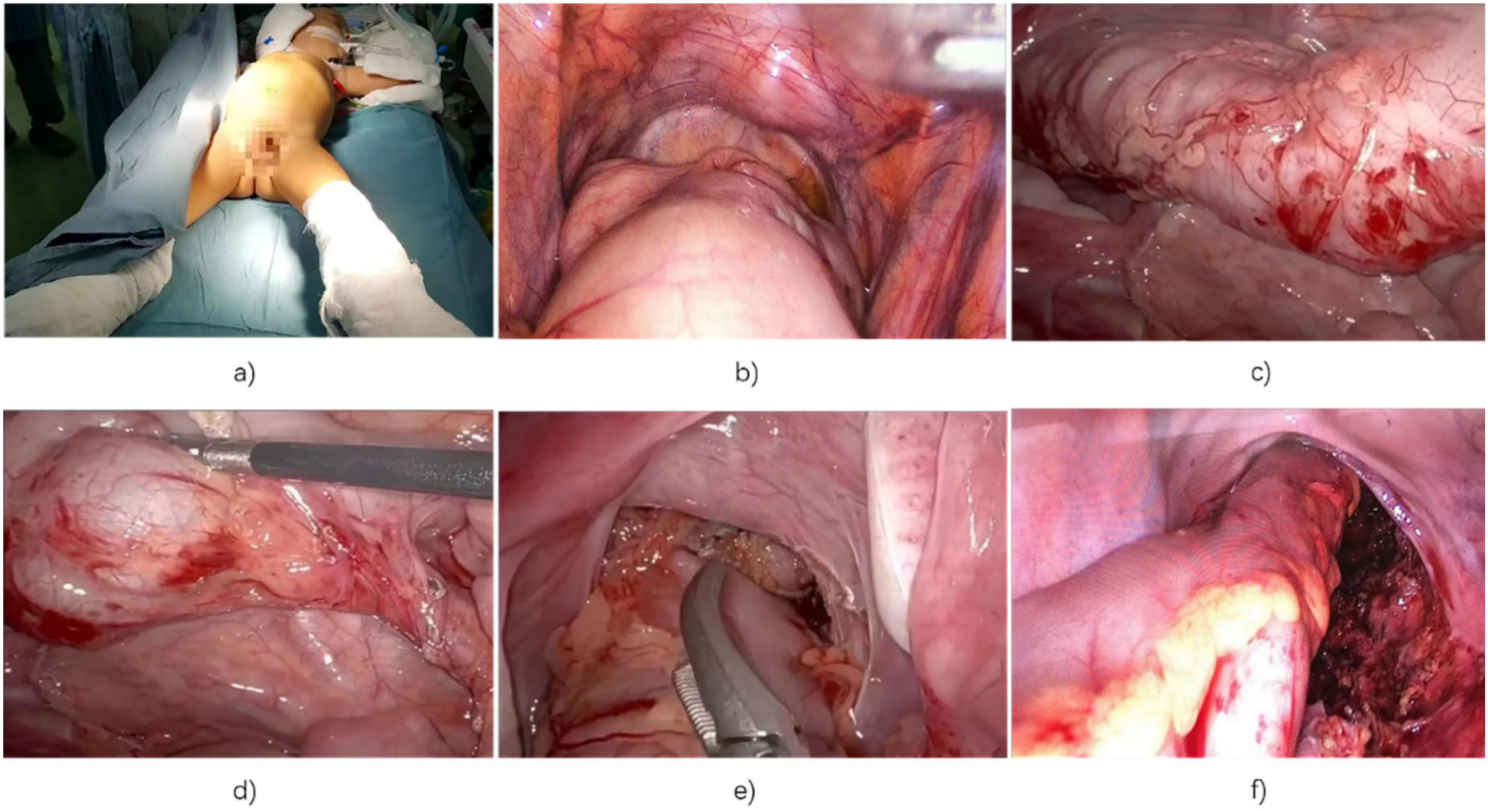
Images of laparoscopic Soave surgery: **(a)** child in the supine position with lower limbs wrapped in sterile towels; **(b)** laparoscopic view showing proximal rectal dilation; **(c)** examination reveals dilation of the descending and sigmoid colon; **(d)** mobilization of the diseased rectum, sigmoid colon, and descending colon; **(e)** dissection of the distal rectum between the serosa and longitudinal muscle; **(f)** postoperative view showing no bleeding, injury, or intestinal torsion.

In cases requiring extended resection, mobilization continued through the splenic flexure, transverse colon, and hepatic flexure, with preservation of the marginal vascular arch. Once sufficient mobilization was achieved, the laparoscopic instruments were removed under sterile conditions. During the transanal phase of the operation, the abdominal area was covered with sterile towels to maintain a sterile environment (Figure 1).

#### 2.5.2 The transanal stage of the laparoscopic modified Soave procedure

Following completion of the abdominal stage of the laparoscopic modified Soave procedure, the operation continued transanally. The patient was moved from the lithotomy position, and an anal retractor was used to expose the distal rectum. Traction sutures were placed 0.5 cm above the posterior dentate line and 2 cm above the anterior dentate line of the rectal wall. At the distal end of the suture, the rectal mucosa was incised with an electric knife through the submucosal layer, and the rectal muscle sheath was dissected along the plane of the anal muscular layer, remaining within the superficial layer of the internal sphincter complex. A 3–4-cm segment of the muscle sheath was preserved.

To prevent internal anal sphincter achalasia and stenosis of the rectal muscle tube, a longitudinal incision was made at the 6 o'clock position of the rectal muscle tube, including the internal anal sphincter. Dissection continued cephalad until the pelvic peritoneal reflection was reached. Under direct vision, the anterior peritoneal reflection was carefully incised, allowing entry into the pelvic cavity. The intestinal segment previously mobilized laparoscopically was then resected transanally. Specimens were sent for pathological examination to confirm complete resection of the aganglionic segment and ensure the remaining bowel was normal.

The proximal colon was examined for adequate blood supply, absence of tension, and lack of torsion, and the operative field was checked for active bleeding. The proximal colon was sutured and fixed to the lateral rectal wall using 4-0 absorbable sutures. Discontinuous anastomosis of the dentate line between the normal proximal colon and rectal mucosa was performed to ensure a tension-free, complete anastomosis. Laparoscopic inspection was then used to confirm that there was no intra-abdominal herniation, hemostasis was adequate, blood supply to the proximal colon was good, and there was no tension or torsion.

**Figure 2:**
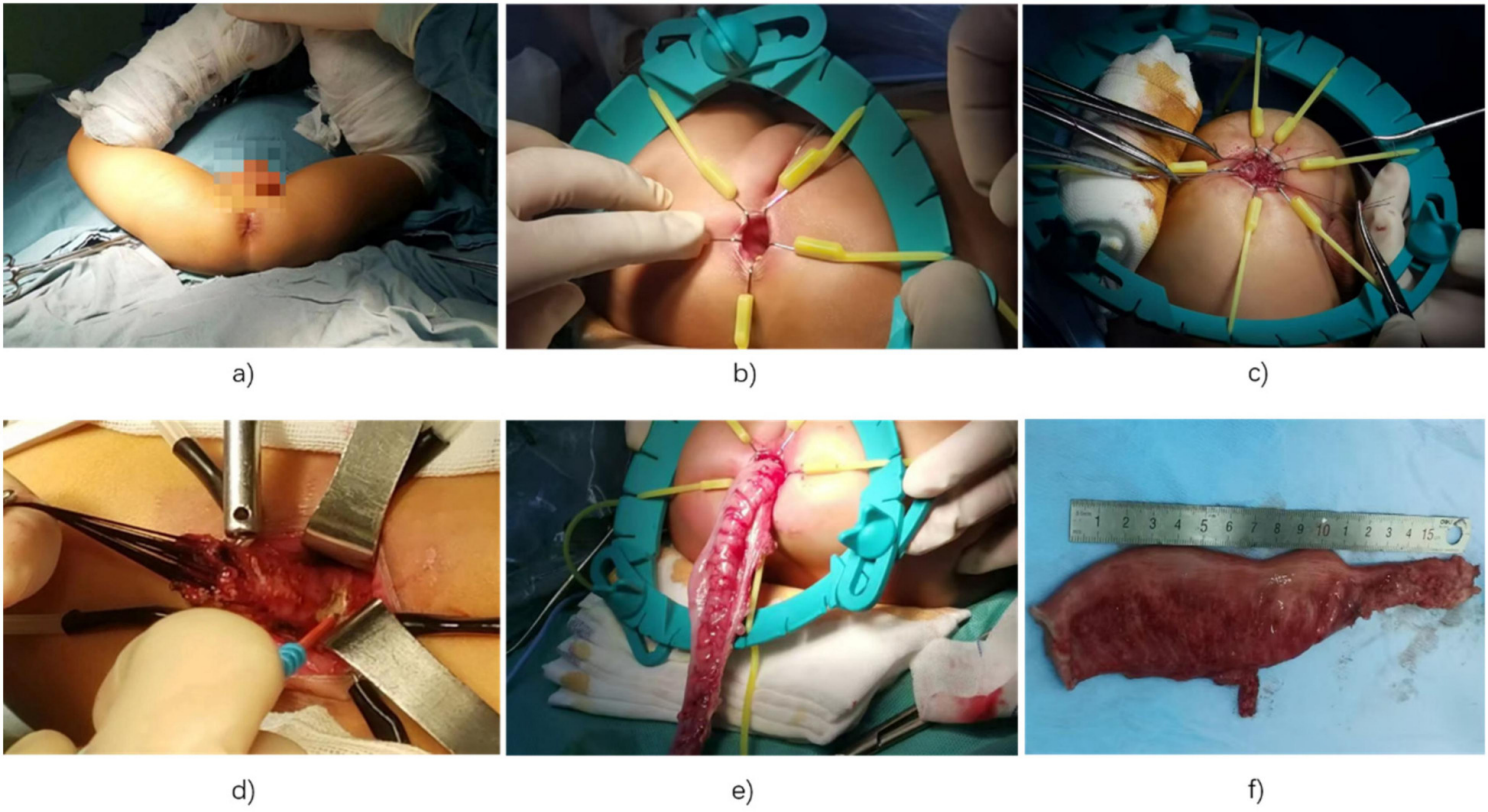
Images of the transanal Soave procedure: **(a)** lower limbs suspended, disinfected, and wrapped in sterile towels; **(b)** anal retractor exposing anal and rectal mucosa; **(c)** traction sutures above the dentate line to mobilize rectal mucosa; **(d)** peritoneal reflection reached and rectal muscle sheath opened, allowing access into the pelvic cavity; **(e)** diseased bowel segment mobilized and resected; **(f)** resection of distal rectal stenosis and colonic dilation.

After removal of the laparoscopic instruments, the abdominal incisions were sutured in sequence. A Vaseline-coated anal canal was inserted and secured. Postoperative care included maintaining anal hygiene to prevent perianal infection (Figure 2).

#### 2.5.3 The transanal Soave procedure

The patient was placed in the lithotomy position, and an anal retractor was used to expose the distal rectum. Traction sutures were placed 0.5 cm above the posterior dentate line and 2 cm above the anterior dentate line of the rectal wall. At the distal end of the suture, the rectal mucosa was incised with an electric knife, dissected through the submucosa, and the rectal muscle sheath was separated along the plane of the anal muscular layer. This dissection remained within the superficial layer of the internal sphincter complex, with 3–4 cm of muscle sheath preserved.

A longitudinal incision was made at the 6 o'clock position of the rectal muscle tube, including the internal anal sphincter, to prevent internal anal sphincter achalasia and stenosis of the rectal muscle tube. Dissection continued in the cephalad direction until the pelvic peritoneal reflection was reached. Under direct vision, the anterior part of the peritoneal reflection was carefully incised to access the pelvic cavity.

The aganglionic bowel segment was further mobilized, and the mesentery was ligated and divided to achieve hemostasis. Dissection continued until normal ganglionic bowel was reached. The mobilized intestinal segment was then delivered and resected through the anus. Specimens were sent for pathological examination to confirm that the aganglionic bowel had been completely resected and that the remaining intestine was normal.

The proximal colon was examined to ensure adequate blood supply and that there was no active bleeding in the surgical field. The proximal colon was sutured and fixed to the bilateral rectal walls using 4-0 absorbable sutures. The normal proximal colonic stump was anastomosed to the rectal mucosa along the dentate line. The anastomosis was confirmed to be tension-free, complete, and without torsion. A Vaseline-coated anal canal was inserted and secured in place. Postoperative care included maintaining anal hygiene to prevent perianal infection.

### 2.6 Statistical analysis

Data were analyzed using SPSS version 24.0. Categorical variables are expressed as frequencies and percentages and compared using the Chi-square test. Continuous variables are presented as mean ± standard deviation and analyzed using the independent-sample t-test. A *P*-value < 0.05 was considered statistically significant.

## 3 Results

### 3.1 Study flow chart

**Figure 3:**
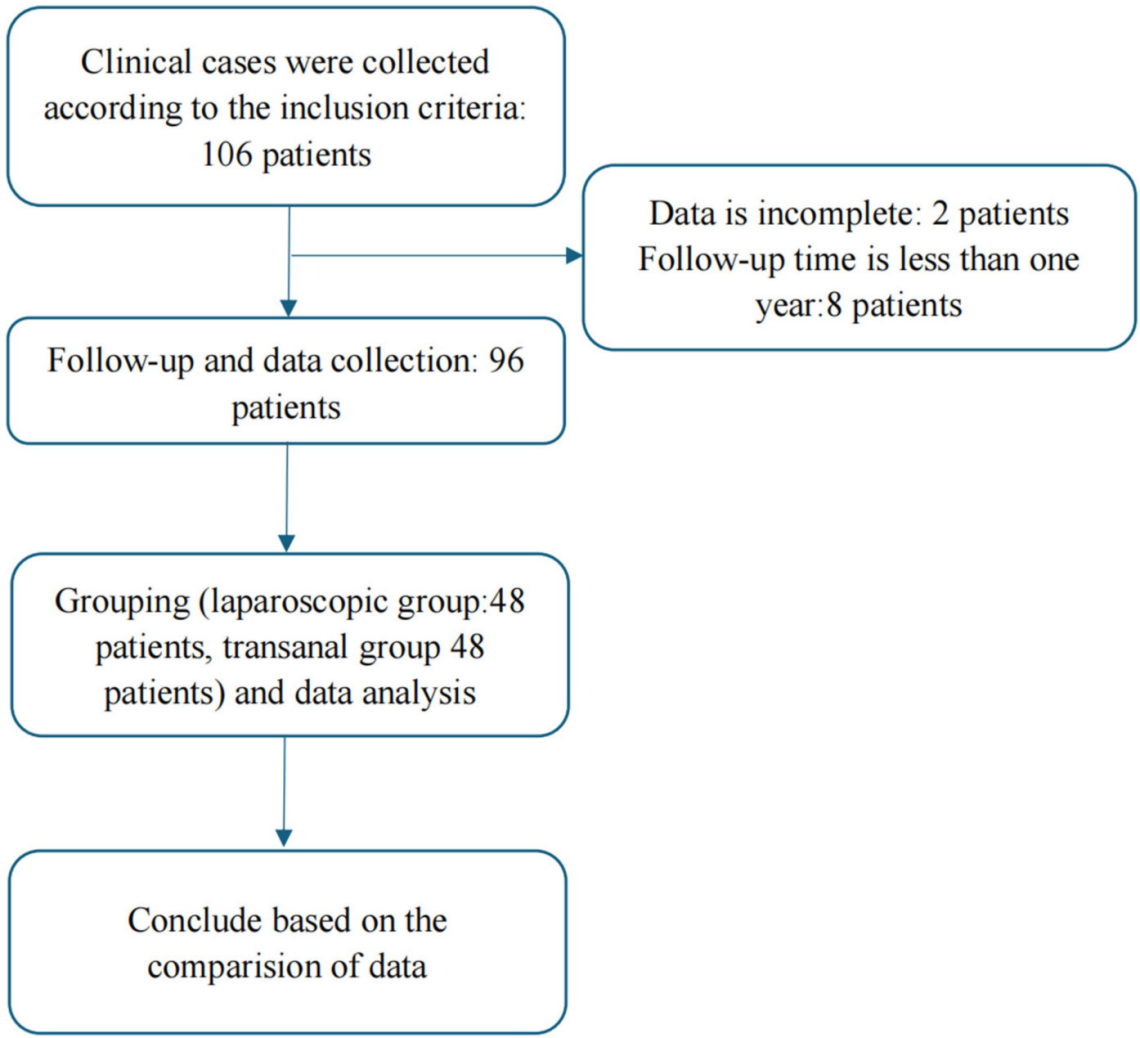
Flowchart description: A total of 106 patients meeting the inclusion criteria were initially enrolled. Among them, eight patients had follow-up periods shorter than 1 year and two patients had incomplete data. Ultimately, 96 patients were included in the analysis. Based on the surgical method, they were divided into two groups for statistical comparison.

A total of 96 patients were included in the study, comprising 57 boys and 39 girls ranging in age from 1 to 103 months, with a mean age of 36.25 ± 14.56 months. Based on the surgical method used, they were divided into two groups for statistical analysis (Figure 3).

### 3.2 Baseline characteristics

A total of 96 children with short-segment HSCR were enrolled, with 48 patients each in the Transanal group and the Laparoscopic group. No statistically significant differences were observed between the two groups regarding gender distribution, age in months, or body weight (*P* > 0.05). All the surgeries were performed by the same surgical team, and no comorbidities were present, indicating that the baseline characteristics were balanced and comparable (Table 1).

**Table 1:** Baseline characteristics between the two groups (mean ± SD).

Total (*n* = 96)	Transanal group (*n* = 48)	Laparoscopic group (*n* = 48)	*P*
Gender			
Female	20	19	0.835
Male	28	29	
Age (month)	33 (19.4, 6.5)	36.5 (20.75, 49.75)	0.996
Weight (kg)	14 (11.25, 16.75)	14 (12, 16.75)	0.996

SD, standard deviation.

### 3.3 Intraoperative and postoperative recovery status

The Laparoscopic group showed significantly shorter surgical time, faster gastrointestinal recovery, and reduced hospital stay duration. However, intraoperative bleeding was greater in the Laparoscopic group compared to the Transanal group (*P* < 0.05) (Table 2).

**Table 2:** Comparison of variables between the two groups (mean ± SD).

Group	Cases	Operative time (min)	Intraoperative blood loss (mL)	Gastrointestinal recovery time (days)	Hospital stay duration (days)
Transanal group	48	235.28 ± 35.68	62.47 ± 8.29	1.78 ± 0.56	14.56 ± 4.02
Laparoscopic group	48	204.59 ± 34.59	75.69 ± 8.56	1.23 ± 0.27	11.16 ± 1.28
*T*	–	4.279	7.686	6.129	5.583
*P*	–	0.000	0.000	0.000	0.000

SD, standard deviation.

### 3.4 Complication rates

Among the 48 patients who underwent the transanal procedure, there were five cases of enterocolitis, one case of wound infection, and one case of intestinal obstruction. In the Laparoscopic group of 48 patients, there was one case of enterocolitis and one case of wound infection. The complication rate was 4.17% in the laparoscopic modified Soave group and 14.58% in the transanal Soave group. However, the difference in complication rates between the two groups was not statistically significant (Table 3).

**Table 3:** Comparison of complication rates between the two groups [*n* (%)].

Group	Cases	Enterocolitis	Wound Infection	Intestinal Obstruction	Total Complication
Transanal group	48	5 (10.42)	1 (2.08)	1 (2.08)	7 (14.58)
Laparoscopic group	48	1 (2.08)	1 (2.08)	0 (0.00)	2 (4.17)
*χ* ^2^	–	–	–	–	3.065
*P*	–	–	–	–	0.080

### 3.5 One-year treatment outcomes

At the 1-year postoperative follow-up, the Laparoscopic group showed a significant reduction in abdominal distension (*P* < 0.05). No statistically significant differences were observed in the other measured outcomes between the groups (Table 4).

**Table 4:** 1-year follow-up outcomes by group [*n* (%)].

Item		Laparoscopic group (*n* = 48)	Transanal group (*n* = 48)	*χ^2^*	*P*
Bowel movements	(≤3/day)	37 (77.08)	33 (68.75)	0.844	0.358
	(>3/day)	11 (22.92)	15 (31.25)		
Bowel control	(Yes)	39 (81.25)	35 (72.92)	0.944	0.331
	(No)	9 (18.75)	13 (27.08)		
Difficulty defecating	(Yes)	5 (10.42)	8 (16.67)	0.801	0.371
	(No)	43 (89.58)	40 (83.33)		
Stool form	(Formed)	42 (87.50)	38 (79.17)	3.011	0.083
	(Unformed)	4 (8.33)	10 (20.83)		
Urination time	(Normal)	47 (97.92)	44 (91.67)	1.899	0.168
	(Abnormal)	1 (2.08)	4 (8.33)		
Abdominal distension	(Yes)	3 (6.25)	14 (29.17)	8.649	0.003
	(No)	45 (93.75)	34 (70.83)		
Fecal soiling	(Yes)	8 (16.67)	11 (22.92)	0.591	0.442
	(No)	40 (83.33)	37 (77.08)		

### 3.6 Multivariate analysis

To eliminate the potential confounding effects of age, gender, and body weight, we performed multivariate analyses. Factors including postoperative complications and follow-up outcomes with a *P*-value < 0.1 in the univariate analysis were included in separate multivariate models, each adjusted for age, gender, and weight. The results indicated that these adjustments did not substantially alter the primary findings.

The total complication rate was not significantly different between the Laparoscopic and Transanal groups (*P* = 0.088, *OR* = 0.239, 95% CI 0.046–1.238). The incidence of postoperative abdominal distension was lower in the Laparoscopic group (*P* < 0.05), but no significant difference was observed in the multivariate analysis of postoperative outcomes (*P* > 0.05). To eliminate potential confounding effects of age, gender, and bod weight, we performed multivariate analyses (Table 5).

**Table 5:** Multivariate analysis of postoperative outcomes.

Item	*P*	OR	95% CI of OR
Total complications	0.088	0.239	(0.046–1.238)
Fecal soiling	0.007	6.161	(1.626–23.348)
Abdominal distension	0.444	1.49	(0.537–4.135)

CI, confidence interval; OR, odds ratio.

## 4 Discussion

### 4.1 Summary of research results

In this study, we analyzed the clinical data of 96 patients, divided into the following two groups: the Laparoscopic group and the Transanal group. Compared with the Transanal group, the Laparoscopic group demonstrated significantly shorter operation time, faster gastrointestinal recovery, and reduced hospital stay duration (*P* < 0.05). Additionally, abdominal distension was significantly improved in the Laparoscopic group (*P* < 0.05). After conducting a multi-factor analysis, this is no longer showed any differences. The overall complication rate did not differ significantly between the Laparoscopic group and the Transanal group (*P* = 0.088, *OR* = 0.239, 95% CI 0.046–1.238). However, the Laparoscopic group demonstrated a significantly lower incidence of fecal soiling (*P* = 0.007, *OR* = 6.161, 95% CI 1.626–23.348). The incidence of abdominal distension was lower in the Laparoscopic group, but the difference was not statistically significant (*P* = 0.444, *OR* = 1.490, 95% CI 0.537–4.135) compared to the Transanal group.

Although the modified laparoscopic Soave procedure showed clear advantages in this study, potential complications such as anastomotic leakage (14, 15), intraoperative bleeding, intestinal obstruction, and wound infection (3, 16) remain concerns. These risks can be minimized through meticulous surgical technique and careful postoperative management (16). The transanal Soave procedure remains a viable option (17), and the choice of surgical method should be individualized based on the patient's condition and disease severity and the available surgical expertise (18, 19).

### 4.2 Comparison with existing literature

The clinical treatment of HSCR primarily involves surgical intervention. The goal is to remove the aganglionic segments of the intestine while preserving normal sphincter function to reconstruct the bowel continuity (20). Surgical approaches can be broadly categorized into traditional open surgery and minimally invasive surgery. Traditional surgery requires laparotomy (21), which results in noticeable surgical scars, a higher risk of complications, and a relatively longer recovery period.

Minimally invasive techniques include laparoscopic-assisted surgery for HSCR (9) and robotic-assisted surgery (21). These approaches offer clear advantages in reducing complications, shortening operative time, and accelerating postoperative recovery, contributing to their widespread clinical adoption. However, robotic-assisted surgery is costly (22, 23), involves a complex setup and dismantling process, and prolongs operative time (24–26). In contrast, laparoscopic-assisted surgery is more practical and accessible (27).

Laparoscopic-assisted radical surgery for HSCR comprises various methods, including the laparoscopic-assisted Soave, laparoscopic-assisted Swenson, and laparoscopic-assisted Duhamel procedures.

Compared to laparoscopic-assisted Swenson surgery (27, 28), the laparoscopic-assisted Soave approach preserves part of the rectal muscle sheath (22), thereby minimizing the impact on anal function and reducing collateral tissue damage from electrocautery during the operation (22, 29). The laparoscopic-assisted Duhamel procedure, however, is relatively complex and time-consuming (30), requiring linear stapling devices (31, 32), which increases the overall cost (33, 34).

Previous studies have demonstrated that the laparoscopic modified Soave procedure offers significant clinical advantages over the transanal Soave procedure in the treatment of HSCR. This minimally invasive approach not only reduces operative time and intraoperative blood loss but also facilitates faster recovery of gastrointestinal function and shortens hospital stay (27, 35). These findings align with prior research emphasizing the benefits of laparoscopic techniques in pediatric surgery (36, 37).

The transanal Soave procedure has become one of the preferred surgical methods for HSCR due to its minimally invasive nature, good preservation of function, and rapid postoperative recovery. It is particularly suitable for children with short-segment lesions (38). When performed by a skilled surgical team, this approach can maximize surgical benefits and minimize risks (39). However, strict adherence to surgical indications is essential. For long-segment or complex cases, laparoscopic assistance should be considered to optimize outcomes. In cases involving total colonic aganglionosis or severe HAEC, the laparoscopic modified Soave procedure should be the preferred method.

### 4.3 Possible mechanisms

It is critical to accurately define the extent of the lesion preoperatively using rectal mucosal suction biopsy, acetylcholinesterase staining, and barium enema. Key technical considerations for the procedure include precisely dissecting along the rectal submucosal plane to avoid injury to the internal sphincter complex; ensuring a tension-free anastomosis when pulling the intestinal segment through, with retention of 3–4 cm of distal rectal sheath; and initiating early postoperative anal dilation starting from postoperative day 14 to effectively prevent anal stenosis.

Furthermore, Georgeson et al. (39) modified the rectal mucosal dissection technique by shifting from a pelvic cavity-based approach to an anal-based separation approach. This adjustment enables more direct access to the surgical field, improves procedural precision, and further minimizes damage to surrounding tissues.

### 4.4 Clinical implications

The laparoscopic modified Soave technique offers superior visualization and operative space compared to the transanal Soave procedure, effectively reducing intraoperative complications and improving long-term outcomes. This technique not only minimizes visible scarring but also lowers the risk of injury to adjacent structures and decreases the risk of early postoperative complications. By employing laparoscopy for muscularis propria or full-thickness biopsy and mesenteric mobilization, the approach avoids the extensive trauma associated with open surgery, resulting in reduced postoperative pain and shorter recovery times.

### 4.5 Limitations and future research

This study had several limitations, including a small sample size, lack of blinding, and a single-center design, which may have reduced the statistical power to detect differences in complication rates. The absence of long-term follow-up limited our ability to fully assess the enduring clinical benefits of the laparoscopic modified Soave technique, including complications such as HAEC. Future research should include larger patient cohorts and extended follow-up periods to validate the clinical advantages of this approach. Comparative studies involving other minimally invasive techniques are also warranted to better define the optimal surgical strategy for HSCR.

## 5 Conclusion

In summary, the laparoscopic modified Soave technique offers improved visualization and surgical control, potentially reducing intraoperative complications and enhancing long-term outcomes. It also decreases the risk of injury to surrounding tissues and lowers early postoperative complication rates. Overall, the laparoscopic modified Soave procedure is an effective treatment for HSCR, providing lower complication rates and faster recovery.

## Data Availability

The raw data supporting the conclusions of this article will be made available by the authors, without undue reservation.
